# Supplementary material for: Experiences of Korean Medicine treatment in patients with Bell’s palsy: A qualitative study protocol
Source: PLoS One. 2025 Oct 8;20(10):e0333488. doi: 10.1371/journal.pone.0333488 (PMC12507202; doi:10.1371/journal.pone.0333488)
Supplement: S1 File — (PDF) [file pone.0333488.s002.pdf]

# Research Plan

# The experiences of Korean medical treatment in patients with facial palsy

Version No. 1.4

Protocol No. FP-QS-2401

Research Institution: Korea Institute of Oriental Medicine

Principal Investigator: Sungha Kim

## 1. Background

Facial palsy is a condition characterized by the weakening or paralysis of facial nerves, resulting in facial muscle paralysis, which causes symptoms such as distortion of the eyes and mouth to one side [1-3]. Acquired facial palsy is a relatively common disorder, with a global annual incidence of 20–32 per 100,000 population and a reported prevalence of 0.12% in South Korea [3-5]. Facial palsy symptoms can lead to severe temporary oral motor dysfunction and even vision impairment due to incomplete blinking, necessitating aggressive treatment in the acute phase [2]. While the prognosis for facial palsy is known to have a complete recovery rate of about 70% [1], cases that do not recover require treatment to improve facial function and management of sequelae such as facial asymmetry, synkinesis, and hypertonicity [6].

In South Korea, facial palsy is an exceptional case where Korean medicine treatment has a higher proportion than Western medicine treatment. In 2021, the number of patients who actually received treatment for facial palsy was 91,251 (49.14%) in Western medicine and 94,428 (50.86%) in Korean medicine [7]. In 2022, 1,339,128 (85.8%) outpatients and 46,129 (14.2%) inpatients were treated at Korean medicine institutions with the diagnosis of facial palsy (G51) [8], ranking 24th in the list of frequent diseases (outpatient) [9]. In Korean medicine, facial palsy is referred to as "Guanwasa" and complex treatments such as acupuncture, electroacupuncture, thread-embedding therapy, pharmacopuncture, herbal medicine, and exercise therapy are performed to restore facial motor function and prevent sequelae [10]. The Korean Medicine Clinical Practice Guideline for Facial Palsy published in 2021 recommends collaborative treatment with acupuncture and Western medicine (A/Moderate), electroacupuncture combined with acupuncture treatment (B/Moderate), and prescribing herbal medicine Gyeonjeongsan in combination with or alongside steroid or acupuncture treatment alone (B/Moderate) [3].

To treat facial palsy, Korean medicine institutions utilize various Korean medicine treatments such as acupuncture, electroacupuncture, herbal medicine, moxibustion, cupping, pharmacopuncture, thread-embedding therapy, Doin exercise therapy, and Chuna manual therapy through the diagnosis of Korean medicine doctors. The effects of Korean medicine treatment have been reported through various quantitative studies such as systematic reviews, RCTs, non-RCTs, and retrospective chart reviews [10,13].

Additionally, qualitative research methods have been used to explore the experiences of inpatient treatment for idiopathic facial palsy and Korean medicine treatment experiences after Western medicine treatment. However, it is difficult to find research that has deeply explored the experiences of facial palsy patients receiving Korean medicine treatment using phenomenological research methods. Therefore, this study aims to explore the impact of Korean medicine treatment on the treatment process of facial palsy patients using qualitative research methods, targeting facial palsy patients who have experienced Korean medicine treatment, to examine in-depth the key elements such as feelings about Korean medicine treatment, goals during the treatment process, and changes in facial palsy status after treatment. The results of this study will be used as subjective evidence for the effectiveness of Korean medicine treatment for facial palsy and as a reference for clinical Korean medicine doctors in their practice.

## 2. Objectives

The central phenomenon to be addressed in this study is ‘the use of Korean medicine treatment for facial palsy’. The central research question is set as ‘How does Korean medicine treatment affect the treatment process of facial palsy patients?’. The sub-questions are as follows:

- How is the decision to use Korean medicine treatment made? (Factors influencing treatment decision)
- What was the effect of Korean medicine treatment on facial palsy? (Subjective effectiveness)
- What did patients experience during the Korean medicine treatment process? (Patient experiences)

The ultimate research objective is to deeply explore the subjective effectiveness and patient perceptions of Korean medicine treatment for facial palsy patients.

## 3. Research Institution Name and Address

- Korea Institute of Oriental Medicine, 1672 Yuseong-daero, Yuseong-gu,

Daejeon 34054, Republic of Korea

## 4. Research Funding Institution

- Korea Institute of Oriental Medicine, 1672 Yuseong-daero, Yuseong-gu, Daejeon 34054, Republic of Korea

## 5. Names and Positions of Principal Investigator, Co-investigators, and Research Staff

| Position | Name | Affiliation and Rank | Role |
|---|---|---|---|
| Principal Investigator | Sungha Kim | Senior Researcher, Korean Medicine Science Research Division, Korea Institute of Oriental Medicine | Result Analysis |
| Co-investigator | Eunbyul Cho | Post-doc, Korean Medicine Science Research Division, Korea Institute of Oriental Medicine | Interview and Result Analysis |

### 5.1 Researchers' Education or Experience in Qualitative Research

| Position | Name | Education Completed | Research History |
|---|---|---|---|
| Principal Investigator | Sungha Kim | Workshop series for qualitative research paper writing (Soongsil University Couple and Family Counseling Research Institute 2013),<br>Seminar on qualitative research methodology applicable to Korean medicine clinical research (Korea Institute of Oriental Medicine, 2014) | Kim S, Chung SE, Han K, Choi S, Lee J-H. Qualitative study on the key elements of obesity counseling in Korean Medicine. J Korean Med. 2023;44(4):1-13. |
| Co- | Eunbyul | Phenomenological qualitative | Cho, E., Lee, DE., Lee, D. et al. |

| | | | |
|---|---|---|---|
| investigator | Cho | research (Education Statistics Research Institute, 2024) | Barriers and future improvements of workplace-based learning in Korean medicine clinical clerkship: perspectives of graduates. BMC Med Educ 24, 566 (2024). https://doi.org/10.1186/s12909-024-05288-3 |

### 6. Research Period

- One year from the date of approval

### 7. Research Subjects

#### 7-1. Research Subjects

- Patients who have experienced Korean medicine treatment for facial palsy

#### 7-2. Inclusion Criteria

- Age 19 or older
- Patients who have experienced Korean medicine treatment for facial palsy at Korean medicine institutions
- Patients whose onset of facial palsy is within 2 years (To avoid recall bias, and based on previous research [16] showing that the accuracy of interviews about experiences more than 2 years after onset is low, we limit to patients within 2 years from the onset date)

#### 7-3. Exclusion Criteria

- Those who do not agree to the research participation consent form and other cases deemed inappropriate by the qualitative research staff

## 8. Expected Sample Size and Calculation Basis

- Saturation is a key principle in determining sample size in qualitative research. In this study, interviews will continue until saturation is achieved. We have set an initial target sample size of 30 participants, but this number may be adjusted based on when saturation is reached. The number of participants in qualitative research does not consider dropout rates because the research is conducted with simple interviews without (non)invasive treatments.

## 9. Recruitment of Participants

- Send cooperation request letters to societies related to facial palsy such as the Korean Acupuncture & Moxibustion Medicine Society and the Korean Medicine Society of Otorhinolaryngology & Dermatology to recommend Korean medicine institutions.
- Contact Korean medicine institutions and post interview participant recruitment flyers to purposively sample participants who have experienced Korean medicine treatment for facial palsy.
- Using the snowball sampling method, ask during the interview who else could be talked to if the researchers hope to interview about this topic, and request the transfer of recruitment documents to people with rich experiences related to the research topic. If the person who receives the document voluntarily wishes to participate in the research, they can contact the research staff's contact information listed on the recruitment document to recruit participants.
- Collect data until each category reaches theoretical saturation and meaningful saturation, and stop recruiting participants when deemed saturated.

## 10. Consent of Participants

- When a person wishing to participate in the interview voluntarily contacts the research staff, the research staff sets a time and place for the interview and meets face-to-face. To protect the personal information of interview participants

and ensure voluntary participation, the research staff (interviewer) obtains consent for research participation face-to-face with the research participant. Before starting the interview, the interviewer shows the participant a written explanation and fully explains the significance of the research to the participant, then obtains a written participation consent. The interviewer signs the consent form as a person delegated by the principal investigator. Two copies of the consent form are made, one is issued to the participant and one is kept by the research team.

## 11. Research Method and Implementation System

- The research will be conducted according to the research progress schedule in the following figure.

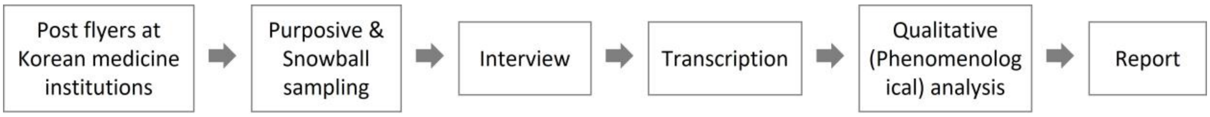


## 12. Data Collection Method and Procedure

### 12.1 Interview Method

- Conduct 1:1 in-depth semi-structured interview between the researcher and individual participants, with the researcher encouraging honest answers and allowing participants to speak freely.
- The main topic of the interview is the experience of Korean medicine treatment for facial palsy, and the interview will be conducted for about 1 hour and 30 minutes to sufficiently collect the participant's responses.
- Interviews between the researcher and participants will be conducted in Korean.
- Interviews will be conducted at a place where the participant feels comfortable, such as a small meeting room or cafe near the participant's residence or workplace.
- If face-to-face interviews are not possible or if the participant prefers an online interview, online interviews will be conducted using the online meeting platform

Zoom.

- Before starting the interview, the researcher starts recording the interview content with the consent of the participant. When the interview is completely finished, the recording is stopped.
- During the interview, the researcher may record non-verbal content such as the participant's behavior or gestures in field notes.
- After the interview, the researcher listens to the recorded interview content repeatedly and transcribes the entire interview content, and stores it together with the records made during the interview.
- Confidentiality is guaranteed for the personal information and statements of the participants.
- The researcher suspends judgment (epoche) during the interview process of collecting raw data to prevent their preconceptions from intervening.

### 12.2 Interview Content

- Stage 1 (Starting Questions): Explain the purpose and precautions of this interview to the participants, introduce the researcher, and create a comfortable atmosphere before the interview. At the beginning of the interview, use the questionnaire to collect basic information about the interview participant (gender, age, characteristics of the residential area, occupation, etc.).
- Stage 2 (Introductory Questions): Interview about the condition at the time of onset, treatment experiences after onset, reasons for visiting Korean medicine institutions, experienced Korean medicine treatments, and points to improve in Korean medicine treatment.
- Stage 3 (Key Questions): Allow participants to freely talk about the reasons for deciding on Korean medicine treatment after the onset of facial palsy, the course of treatment, and their treatment experiences.
- Stage 4 (Concluding Questions): Check if there is anything that was not said during the interview process, and get recommendations for people to interview related to this topic for snowball sampling.

## 13. Effectiveness Evaluation Criteria and Methods

- Not applicable

## 14. Safety Evaluation Criteria and Evaluation Methods

- Not applicable

## 15. Data Analysis Method

### 15.1 Transcription

- After the interview is completed, the researcher listens to the recorded audio file of the interview content in the research room and writes a detailed script.

### 15.2 Analysis and Interpretation Method of Survey Results

- The researcher transcribes the recorded interview content and repeatedly listens to the original data while comparing it with the content of the original data, confirms unclear parts with the participant by meeting directly or by phone, and refers to field notes to ensure the accuracy of the interview content.
- Analyze, summarize, and draw conclusions and suggestions using the phenomenological research method of qualitative research [15], and write a paper.

### 15.3 Expert Consultation

- Receive expert consultation at all stages of the research, including research design, questionnaire development, interview conduct, result analysis, and paper writing, to increase the completeness of the research.

### 15.4 Ensuring Reliability and Validity of the Research

- To ensure the reliability and validity of the research, conduct triangulation of data and researchers to cross-verify the research results. Data triangulation involves collecting various data such as interview transcripts and the researcher's field notes. For researcher triangulation, while the research staff primarily conducts interviews and analysis, the principal investigator and qualitative research experts provide consultation and supervision throughout the research process to exclude the researcher's bias as much as possible.
- The researcher continuously strives to suspend judgment (epoche) to prevent their preconceptions from intervening in the interview process of collecting raw data.

**16. Anticipated Side Effects and Precautions and Measures**

- Not applicable

**17. Discontinuation and Dropout Criteria**

- During the research, subjects can voluntarily withdraw from qualitative research participation at any time, or if deemed inappropriate for qualitative research progress by the researcher's judgment, they may be dropped out.

**18. Risks and Benefits for Participants**

**18-1. Risks for Participants**

- There are no anticipated risks, and the research is conducted considering the mental and psychological discomfort of the participants.

**18-2. Benefits for Research Subjects**

- Not applicable. As compensation for research participation, a compensation of 100,000 KRW per interview is provided.

## 19. Safety Measures and Personal Information Protection Measures for Participants

### 19-1. Confidentiality

- Personal information is anonymized when creating scripts to make personal identification impossible.
- Although personal information of research participants (gender, age, characteristics of residential area, occupation, experience of employment status change due to facial palsy onset, date of facial palsy onset, current facial palsy treatment situation, Western medicine currently taken due to underlying diseases, total period of taking herbal decoctions) is collected, records that can identify individuals are anonymized by assigning management numbers and stored on computers as password-protected files.
- Research-related materials, including notes and journals written during interviews, are stored in a locked data storage room to ensure strict confidentiality and protection.
- Collected personal information is stored for 3 years from the end date of the research and then permanently deleted.
- Records that can identify research subjects will be kept confidential, and the identity of research subjects will be maintained in a confidential state even if research results are published.

### 19-2. Storage of Research-Related Documents and Data

- Research-related documents are stored in a locked data storage room to maintain security.
- Data acquired after the end of the research is stored separately in accordance with IRB regulations.
- After completing the results report, a storage manager is designated to preserve research-related documents for 3 years from the end date of the research, and after the storage period has passed, they are destroyed using a

shredder.

- Transcription-related materials such as transcripts and audio files are stored on the researcher's computer as encrypted files for 3 years from the end date of the research, and when the storage period ends, the files are permanently deleted.

**19-3. Measures for Protecting Subject Safety and Compensation Rules for Victims**

- This study is qualitative research and no safety issues for subjects are anticipated. However, if injury occurs directly related to the research procedures according to the protocol, this research team will take the best medical measures for this.

**20. References**


1. Peitersen E. Bell's palsy: the spontaneous course of 2,500 peripheral facial nerve palsies of different etiologies. Acta oto-laryngologica. 2002;122(7):4–30.
2. Baugh RF, Basura GJ, Ishii LE, Schwartz SR, Drumheller CM, Burkholder R, et al. Clinical practice guideline: Bell's palsy. Otolaryngology–Head and Neck Surgery. 2013;149(3_suppl):S1–27.
3. Korean Acupuncture & Moxibustion Medicine Society. Clinical practice guideline of Korean medicine for Facial palsy. National Institute for Korean Medicine Development; 2021.
4. Lorch M, Teach SJ. Facial nerve palsy: etiology and approach to diagnosis and treatment. Pediatric emergency care. 2010;26(10):763–9.
5. Chang YS, Choi JE, Kim SW, Baek SY, Cho YS. Prevalence and associated factors of facial palsy and lifestyle characteristics: data from the Korean National Health and Nutrition Examination Survey 2010–2012. BMJ open. 2016;6(11):e012628.
6. Vakharia K, Vakharia K. Bell's palsy. Facial Plastic Surgery Clinics. 2016;24(1):1–10.
7. Yoon HC. A study on the characteristics of patients with facial palsy treated with

Korean medicine–Based on the Korean national health insurance statistical yearbook. Journal of Korean Medicine. 2023;44(2):10–9.
8. Health Insurance Review & Assessment Service. https://opendata.hira.or.kr/op/opc/olap3thDsInfoTab1.do
9. Health Insurance Review & Assessment Service. https://opendata.hira.or.kr/op/opc/olapHifrqSickInfoTab1.do
10. Lee SE, Yoon HJ, Ko WS. Research trends on non-surgical treatment of peripheral facial paralysis sequelae. J Korean Med Ophthalmol Otolaryngol Dermatol. 2018;31(4):59-73.
11. Ministry of Health and Welfare. Pilot project to apply herbal medicine to health insurance. 2020.
12. National Institute for Korean Medicine Development. 2022 Korean Medicine Utilization Survey Basic Report - General Public. 2023.
13. Bae HB, Yoon HJ, Ko WS. A retrospective study of facial paralysis sequelae for Korean medical treatment. J Korean Med Ophthalmol Otolaryngol Dermatol. 2019;32(1).
14. Hennink MM, Kaiser BN, Marconi VC. Code Saturation Versus Meaning Saturation: How Many Interviews Are Enough? Qual Health Res. 2017 Mar;27(4):591–608.
15. Kim B.H., et al. A Comparison of Phenomenological Research Methodology. Journal of Korean Academy of Nursing. 1999: 29(6); 1208-1220.
16. Stormon, N., & Sexton, C. (2023). Parental recall bias in observational studies: Child dental service use. International Journal of Paediatric Dentistry, 33(5), 450-456.
