# Supplementary material for: Experiences of Korean Medicine treatment in patients with Bell’s palsy: A qualitative study protocol
Source: PLoS One. 2025 Oct 8;20(10):e0333488. doi: 10.1371/journal.pone.0333488 (PMC12507202; doi:10.1371/journal.pone.0333488)
Supplement: S2 File — (PDF) [file pone.0333488.s003.pdf]

S2 File

Interview guide

Introduction

Thank you for making time to participate in this study. The researchers are two Korean Medicine doctors and a nurse; we are not directly involved in your treatment. Please feel free to tell us about your experience with Korean Medicine treatment after Bell's palsy. There are no right answers. The duration of this interview will be approximately 60–90 minutes. Your personal information will be kept confidential. The interview will be audio recorded, and you may ask to stop the recording during the interview. Do you have any questions before we begin?

Background information and treatment history

- Which gender do you most identify?
- What is your current age?
- Among the following options, please select the one that most closely describes the area where you live: city—residential area, city—commercial area, city—industrial area, farm village, fishing village, or mountain village.
- What is your profession?
- Has your employment status been affected by Bell's palsy?
- When was the onset date of your condition?
- What is the current state of your Bell's palsy treatment?
- Are you currently taking any medications for an underlying medical condition other than Bell's palsy?
- What Korean Medicine treatment have you received to date for Bell's palsy?

Experience with Korean Medicine treatment for Bell's palsy

- Please tell me your overall impression (feeling) when you think about your Korean Medicine treatment.
- What did you find impressive about the treatment?
- What were your goals when you decided to undergo Korean Medicine treatment? How did you feel during the treatment, and how did you feel after it?
- Did you talk to anyone before, during, or after the Korean Medicine treatment?
- Did you notice any changes in your facial condition after you received the treatment?
- Was there anything easy, difficult, good, or bad about your treatment?
- Is there anything else you would like to add?

These are all the questions we have. If you have any other comments, please feel free to tell us. Thank you for your participation.
