# Supplementary material for: Experiences of Korean Medicine treatment in patients with Bell’s palsy: A qualitative study protocol
Source: PLoS One. 2025 Oct 8;20(10):e0333488. doi: 10.1371/journal.pone.0333488 (PMC12507202; doi:10.1371/journal.pone.0333488)
Supplement: Attachment — Submitted filename: Response to Reviewers_2nd round.docx [file pone.0333488.s006.docx]

[Sep 14, 2025]

Dear Editor:

We are pleased to submit a revised version of our manuscript entitled “**Experiences of Korean Medicine treatment in patients with Bell’s palsy: a qualitative study protocol**” for publication in *PLOS ONE*.

We appreciate the editor’s comment regarding the similarities to Yoon et al. (2022). We have carefully revised the manuscript to clarify the distinctions between our study and the prior work.

In terms of the research question, Yoon et al. (2022) concentrated on the process of choosing Korean Medicine after Western treatment for facial palsy in general, whereas our protocol focuses on the lived experiences and meanings of Korean Medicine treatment specifically among patients with Bell’s palsy.

From a methodological perspective, the prior study used grounded theory to build a paradigm model of treatment selection, while we apply Colaizzi’s descriptive phenomenological approach to capture the essence of patient experiences.

Concerning participants, the earlier study involved a small sample of ten individuals with mixed etiologies such as cholesteatoma, trauma, Ramsay Hunt syndrome, and central facial palsy, limiting its ability to reflect Bell’s palsy–specific perspectives; our study targets a more homogeneous group of Bell’s palsy patients within two years of onset, with a larger sample until saturation.

Finally, with respect to the expected differences in outcomes, the earlier study highlighted treatment decision-making and suggestions for improving KM, while our work explores motivations, perceptions, and recovery experiences, aiming to inform patient-centered care in Bell’s palsy.

In the revised ***Introduction***, we have added the following sentence:

*“Another qualitative study applied grounded theory to focus on the process of choosing KM after CM treatment, but it mixed various etiologies of facial palsy—including cholesteatoma, trauma, Ramsay Hunt syndrome, and central facial palsy—thus lacking a dedicated exploration of Bell’s palsy–specific patient experiences (Yoon et al., 2022)”*

We carefully reviewed all references to ensure accuracy and confirm that none of the cited works have been retracted. During this process, we identified one reference (Morrow et al., 2015) with incomplete bibliographic details and therefore removed it from the revised manuscript.

We appreciate your consideration of our revised manuscript.

Best regards,
Sungha Kim, KMD, Ph.D.
KM Science Research Division, Korea Institute of Oriental Medicine
(34054) 1672 Yuseong-daero, Yuseong-gu, Daejeon, Republic of Korea
Tel: 82-(0)42-868-9345
E-mail: bozzol@kiom.re.kr

**Dear Reviewers,**

Thank you for your valuable time and effort in reviewing our manuscript. We believe that the manuscript has been improved based on your thoughtful comments. Changes have been marked as track changes in the manuscript and described with responses.

**Reviewer #1**

**[Comment]**

This is a well-structured and thoughtfully designed qualitative protocol that fills a crucial gap in understanding patient experiences with Korean Medicine for Bell’s palsy. The rationale is compelling, methods are rigorous, and the authors have carefully addressed potential biases, reflexivity, and ethical considerations.

Notably commendable elements include:
- Integration of both purposive and snowball sampling with bias mitigation strategies
- Explicit measures to support participant comfort, authenticity, and emotional safety
- Use of validated qualitative methods and commitment to data saturation
- Clear documentation and transparency in data sharing plans

Minor suggestions:
- Consider proofreading again for small formatting inconsistencies in references or file labeling (e.g., “mMedicine” in one title).
- You may consider expanding dissemination plans for international audiences through multilingual abstracts or summary materials.

I support the acceptance of this study protocol.

**Response:** We sincerely thank the reviewer for the generous and encouraging evaluation of our study protocol. Regarding the minor suggestions, we carefully proofread the references and corrected formatting inconsistencies. In the revised manuscript, we modified the ***Dissemination*** section as follows:
*“We intend to disseminate the findings of this study through academic journals, seminars, and conference presentations, including the preparation of multilingual abstracts to reach international audiences.”*
We believe this addition will clarify our plan to make the study findings more accessible beyond domestic contexts.

**Reviewer #2**

**[Comment]**

This revised protocol presents a timely and well-structured qualitative study exploring the experiences of patients with Bell’s palsy undergoing Korean Medicine (KM) treatment. The authors have thoughtfully addressed all prior reviewer comments, resulting in an improved and more transparent protocol.

**Response:** We sincerely appreciate the reviewer’s positive evaluation and encouragement.
